# Can PPSV-23 Vaccine Impact Exacerbations of Chronic Cough Symptoms and Medication Use in Younger Adult Asthmatics? A Clinical Question That Needs Answering

**DOI:** 10.3390/vaccines10020219

**Published:** 2022-01-30

**Authors:** Alessandra M. Lanz, Esther Chartrand, Claudia P. Eisenlohr, Miguel J. Lanz

**Affiliations:** Allergy, Asthma & Sinusitis, AAADRS Clinical Research Center, Coral Gables, FL 33134, USA; alessandramlanz@gmail.com (A.M.L.); echartrand365@gmail.com (E.C.); claudia.eisenlohr@gmail.com (C.P.E.)

**Keywords:** asthma exacerbation, chronic cough, systemic steroids, exhaled nitric oxide, serum eosinophils, total IgE, PPSV23, vaccine responsiveness, vaccination

## Abstract

Symptoms of chronic cough (CC) from the airways are commonly treated with antibiotics, antitussives, bronchodilators, and steroids. There is a wide variability in treatment response, dependent on the exact cough etiology. Our case-series study was composed of 71 nonsmoking adults, 59 females, mean age 43 (±21) years, with a history of CC-asthma and history of ≥2 exacerbations/year requiring systemic steroids and/or antibiotics. All had decreased *Streptococcus pneumoniae* antibody titers, with a mean average of 3 of 23 normal serotypes and were subsequently vaccinated with PPSV-23. Pre- and post-12-month vaccination questionnaires were administered, and 35 (54%) reported both decreased CC symptoms and asthma medication use. Baseline comparisons to those with no change in CC symptoms or asthma medication use revealed significantly lower exhaled nitric oxide (FeNO) levels (17 ± 10; 62 + 40 ppb), serum eosinophils (192 ± 156; 280 ± 166/mcL), and total IgE (132 ± 167; 275 ± 290 IU/mL) in those with improvement post-vaccination. Higher baseline symptoms scores for upper respiratory infections as a trigger to their CC (* *p* > 0.05) were found in those responding to PPSV-23. These data reveal a subset of asthma in younger adults, <65 years, with significantly decreased *S. pneumoniae* antibody titers with less CC symptoms and asthma medication use for exacerbations after PPSV-23 vaccination.

## 1. Introduction

Serious pneumococcal infections have been documented to be an increased risk factor for asthmatics even when controlling other risk factors for invasive disease and cigarette smoking [1,2]. Additionally, the serotype response to 23-valent pneumococcal vaccine (PPSV-23) is decreased in asthmatics compared to controls [3,4]. Unfortunately, the use and adherence of PPSV23 vaccination for asthmatics is poor [5], especially under 65 years of age.

Asthma is a heterogeneous respiratory disease with multiple phenotypes arising from interactions between genetics, infections, and environmental exposures [6]. Symptoms of asthma can be varied, ranging at times between asymptomatic periods to episodes with symptoms that can lead to exacerbations. Common symptoms are chronic cough, exercise intolerance, chest tightness, and shortness of breath. One measure of asthma control is defined clinically [6] as asthma symptom-reporting and reliever use less than twice per week. Asthma impairment is the assessment of the frequency and intensity of symptoms that a patient is experiencing. Recurrent treatment with systemic steroids for impairment and loss of asthma control can progress to increased secondary side effects. The goal of treatment as per GINA/NAEPP asthma guidelines is to personalize medical treatment to the patient’s asthma symptoms and objective measurements of respiratory tests [6].

Research advances and real-world studies in asthma management have shown various observable patient phenotypes in asthmatics influenced by underlying endotypes, or inflammatory milieu, which are commonly triggered by infectious viral/bacterial antigens, allergens, or pollutants. One paradigm for endotype differences in chronic asthma is Th-2 vs. Th-1 cytokines [7]. Th-2 driven asthma is mainly propagated by IL-4, IL-5, and IL-13 and seen with biomarker elevations of IgE, eosinophils, and FeNO, respectively [8]. As a result, there are various asthma treatment modalities, including specific biologic therapies. Patients with Th-1 asthma have increased cytokines, like Th-17 that do not elevate Th-2 biomarkers and which are triggered by respiratory infections, amongst others [9]. At present, beyond a few specific in vitro tests for identification, there are no easily accessible clinical biomarkers or specific asthma treatments for this Th-1 population.

Common comorbidities, such as rhinosinusitis, gastroesophageal reflux disease (GERD), and vocal cord dysfunction are some diagnoses that can mimic asthma and even make its control more challenging, when not addressed. In US adults of all ages, asthma, upper airway cough syndrome (UACS), known as post-nasal drip syndrome, and GERD are the three most common causes of chronic cough. CC is likely due to asthma, UACS, GERD, and non-asthmatic eosinophilic bronchitis in 92% of patients who are non-smokers, not taking an ACE inhibitors, and have a normal chest X-ray [10]. CC can be the sole presentation in patients with asthma. Recurrent episodes of CC symptoms of >8 weeks can present in patients, who have not had its underlying cause addressed. As asthma specialists, allergist/immunologists have training investigating the immunologic state of asthmatics for immune dysregulation, including functional antibody deficiency, one of the common immune deficiencies.

We proposed to evaluate asthma control and exacerbation risk by the patient report rate of symptoms and medication use in exacerbation-prone asthmatics with high frequency of systemic steroid bursts and/or antibiotic use. Second, we will attempt to identify baseline objective measures and symptom reports to elucidate differences in treatment responses between asthma endotypes, such as infection-prone (Th-1) versus atopic-driven (Th-2) asthmatics, with subsequent exacerbation outcomes.

## 2. Materials and Methods

We conducted a 5-year chart review from an allergy/asthma/immunology clinic for patients with a history of cough-variant asthma and history of ≥2 exacerbations requiring systemic steroids and/or antibiotics in the previous year.

All patients were first-time referrals to the specialist clinic for medical evaluation of their CC/asthma due to their high frequency of respiratory exacerbations requiring systemic medications. Patient questionnaires were given pre- and post-treatment to capture the symptom frequency and medication use, specifically systemic steroids and antibiotics, for their respiratory exacerbations in the prior year and subsequent year. Specific pre- and post- questions included the frequency of asthma exacerbations as the “number of asthma flare-ups” and “more, or same, or less than prior 12 months”. The frequency of “use of antibiotics for asthma flare-ups” and “use of oral/intramuscular steroids (prednisone/methylprednisolone) for asthma flare-ups “were collected with the amount captured as “more, or same, or less than prior 12 months”.

After an extensive history and physical examination by the asthma specialist, all patients had testing for respiratory allergies and immunological evaluations. Baseline allergy evaluations consisted of allergy prick skin testing to environmental/food allergens and laboratory IgE-allergen specific tests. All had baseline respiratory testing consisting of spirometry and FeNO measurements, prior to medication use. Baseline immunology testing consisted of CBC with differential, absolute total immunoglobulins, and specific antibody titers responses to Streptococcus pneumoniae.

All patients were evaluated and treated as per the standard of medical care for CC and asthma. Those with uncontrolled GERD, present or past history of smoking, ACE-inhibitor use, underlying cardiac or airway anatomical conditions, undiagnosed weight loss, unexplained fevers, chronic peripheral edema, or abnormal chest films were excluded from the study. This study followed current clinical standard medical care which was reviewed and exempted by the ethics/IRB committee of the Salus Institutional Review Board.

## 3. Results

Our case-series study consisted of 71 non-smoking adults, 59 females, mean age 43 ± 21 years. At baseline, almost 90% were found to have allergen sensitization to ≥1 allergen by prick skin testing and laboratory allergen specific-IgE levels. The mean FeNO level for all the patients was 38 ± 35 ppb, which is elevated (normal < 25–30 ppb) and consistent with airway inflammation. The mean spirometry FEV1 was 84 ± 26%. These patients were found to have decreased *S. pneumoniae* antibody titers with a mean average of 3.4 ± 2.6 of 23 serotypes (normal > 1.3 mcg/mL). The most common Pneumococcal serotypes that were low or absent of antibody responses were types 12 (12 F), 22 (22 F), 68 (9 V). Mean white blood corpuscles were 6600 ± 1658/mcgL, serum neutrophils 3938 ± 2460/mcgL (58%), serum eosinophils 241 ± 178/mcgL (4%), and immunoglobulins IgG 1044 ± 265 mg/dL, IgA 223 ± 95 mg/dL, IgM 175 ± 50 mg/dL, and IgE 195 ± 239 IU/mL were all in the normal ranges. 

Patients were vaccinated with PPSV23 (Pneumovax-23, Merck Sharp Dohme Corp., West Point, PA, USA) due to low titers to *S. pneumoniae* serotypes. The questionnaires given pre- and post-PPSV23 vaccination (mean +12 months) were to capture the symptom frequency and medication use of systemic steroids and antibiotics for their respiratory exacerbations in the prior and subsequent year. Of the 65 patients who returned completed post-vaccination questionnaires, 35 (54%) reported both decreased respiratory exacerbations and use of systemic steroids/antibiotics in the year following PPSV23 vaccination. In the following year post-vaccination, these patients had decreased urgent care/ER visits and unscheduled health care visits for exacerbations, in addition to less use of rescue medications.

Comparisons between responders with fewer asthma exacerbations and need for systemic medications with those that did not improve post-PPSV23 vaccination were assessed by their baseline characteristics. At baseline, significantly lower FeNO (17 ± 10, 62 ± 40 ppb * *p* > 0.05), serum eosinophil counts (192 ± 156, 280 ± 166/mcL * *p* > 0.05), and total IgE (132 ± 167, 275 ± 290 IU/mL, * *p* > 0.05) were found in those patients who responded clinically and by questionnaires post-PPSV23 vaccination. Responders had significantly less allergen sensitization by skin testing or allergen-specific IgE than those who did not respond, 81% vs. 97%, * *p* > 0.05) (Table 1). Triggers to respiratory symptoms were reported to be more frequent upper respiratory infections in those who responded to PPSV23 compared to those that did not (* *p* > 0.05). No differences were found at baseline with spirometry measurements, WBC, neutrophils, IgG, IgA, IgM, or number of antibody titers between groups.

## 4. Discussion

Our study aimed to identify clinical asthma phenotypes related to differences in subsequent treatment responses as measured by their exacerbation rate. First, those with higher FeNO, serum eosinophils, total IgE levels, and allergen sensitivity, consistent with Th-2 asthma, continued with a higher frequency of exacerbations. Those with lower Th-2 markers of asthma did have lower frequency of exacerbations post-PPSV23 vaccination. Additionally, these real-world data are unique in the literature because our subset of adult asthmatics with low specific antibody titers to *S. pneumoniae* had reductions in asthma exacerbation and morbidity with decreased systemic steroid and antibiotic use, after PPSV23 vaccination. Prior studies have shown similar findings in the pediatric asthma population [11,12].

Asthma is an independent risk factor for invasive pneumococcal disease [1,2]. There are specific pneumococcal serotypes that have been documented to have increased invasive potential [13,14], like serotype 68 (9 V) that we found significantly decreased titers to in our study population. Similarly, a smaller study by Laratta [4], in a case series of 17 adult asthmatics taking high-dose inhaled or oral steroids showed almost half with low antibody titers to serotype 68 (9 V) among five serotypes collected. The other two serotype titers in our data to be most decreased, serotypes 12 (12 F) and 22 (22 F), are considered in invasive infections as other serotypes. However, specific pneumococcal serotypes causing invasive infection may not be directly related to increased asthma exacerbations, either. Nevertheless, larger randomized-controlled specific serotype studies into possible causal relationships to noninvasive infections and asthma exacerbations are sorely needed [3]. The inflammatory milieu of the airways caused by respiratory infection could be significantly different than from an invasive infection.

Limitations to our study are its case-series design without a control group, single-site from an asthma specialist clinic, and use of a subjective patient questionnaire. Prospective, randomized-controlled designed studies, with multiple sites, would be strongly recommended. Future directions for studies in younger adults with frequent asthma exacerbations are recommended to further evaluate the immunologic response and treatment of specific antibody deficiencies, like to *S. pneumoniae*. Although present guidelines do recommend pneumococcal vaccinations in asthmatics ≥65 years of age [15], this data potentially hypothesizes an asthma subtype under 65 years of age, which may benefit from vaccination to reduce asthma impairment and risk. Identification of adult asthmatics with low FeNO, eosinophils, and total IgE could provide the beginnings of a targeted therapeutic approach with pneumococcal vaccination to reduce asthma exacerbations.

## 5. Conclusions

In evaluating young adult asthmatics, less than 65 years, with recurrent asthma exacerbations requiring frequent systemic steroid bursts and/or antibiotics, evaluation for low functional antibody responses to *S. pneumoniae* can be considered. Identification of these asthmatics, who have lower FeNO, eosinophil counts, and total IgE could benefit from this immune evaluation to reduce exacerbations. This identification of low specific antibody responses to *S. pneumoniae* serotypes may provide an avenue of treatment to reduce respiratory exacerbations in this precise asthma phenotype. Further studies are needed to answer the clinical question if correction of low pneumococcal titers can impact symptoms and medication use in asthmatics.

## Figures and Tables

**Table 1:** Baseline characteristics between responders vs. non-responders.

Patient Parameters	Responders	Non-Responders	Stat Significance
Demographics			
Number	35	30	
Age, mean (y)	44 ± 19	42 ± 20	n.s.
Females (%)	89	76	
Positive PST/specific IgE (%)	81	97	* p < 0.05
FeNO (ppb), mean	17 ± 10	62 ± 40	* p < 0.05
FVC%, mean	87 ± 8	83 ± 15	n.s.
FEV1%,mean	86 ± 9	83 ± 16	n.s.
FEF25–75%, mean	89 ± 28	83 ± 29	n.s.
PEFR%, mean	88 ± 12	91 ± 15	n.s.
Laboratory tests, mean			
WBC (×10^3^/mcL)	6.5 ± 2.8	7.0 ± 2.7	n.s.
Neutrophils, serum (/mcL)	3923 ± 1671	3947 ± 2866	n.s.
Eosinophils, serum (/mcL)	192 ± 156	280 ± 166	* p < 0.05
IgE total (IU/mL)	132 ± 167	275 ± 290	* p < 0.05
IgG total (mg/dL)	1005 ± 343	1097 ± 331	n.s.
IgA total (mg/dL)	208 ± 85	245 ± 92	n.s.
IgM total (mg/dL)	193 ± 56	153 ± 41	n.s.
*S. pneumoniae* titers (nl, >1.3 mcg/mL)	3.0 ± 2.6	3.9 ± 2.5	n.s.

Two-tailed *t*-test * *p* < 0.05; n.s. = no sig.

## Data Availability

Not applicable.
